# Contribution of Infrared Spectroscopy to the Understanding of Amyloid Protein Aggregation in Complex Systems

**DOI:** 10.3389/fmolb.2022.822852

**Published:** 2022-04-08

**Authors:** Diletta Ami, Paolo Mereghetti, Antonino Natalello

**Affiliations:** ^1^ Department of Biotechnology and Biosciences, University of Milano-Bicocca, Milano, Italy; ^2^ Bioinformatics Consultant, Arquata Scrivia, Italy

**Keywords:** amyloid, sub-cellular, microspectroscopy, proteins, infrared spectroscopy

## Abstract

Infrared (IR) spectroscopy is a label-free and non-invasive technique that probes the vibrational modes of molecules, thus providing a structure-specific spectrum. The development of infrared spectroscopic approaches that enable the collection of the IR spectrum from a selected sample area, from micro- to nano-scale lateral resolutions, allowed to extend their application to more complex biological systems, such as intact cells and tissues, thus exerting an enormous attraction in biology and medicine. Here, we will present recent works that illustrate in particular the applications of IR spectroscopy to the *in situ* characterization of the conformational properties of protein aggregates and to the investigation of the other biomolecules surrounding the amyloids. Moreover, we will discuss the potential of IR spectroscopy to the monitoring of cell perturbations induced by protein aggregates. The essential support of multivariate analyses to objectively pull out the significant and non-redundant information from the spectra of highly complex systems will be also outlined.

## Introduction

The average life expectancy has been increasing at a rapid rate in industrialized countries, but unfortunately with a longer life expectancy comes a higher chance of developing a neurodegenerative disease, such as Alzheimer’s, Parkinson’s, Huntington’s, and others. A common key molecular pathway implicated in different neurodegenerative and non-neurodegenerative amyloid diseases is the misfolding and aggregation of proteins that cause cellular toxicity and contribute to cell proteostasis collapse, a key hallmark of cell aging (Chiti and Dobson, 2006; Chiti and Dobson, 2017; Moreno et al., 2019; Santra et al., 2019). In this regard, despite significant efforts aimed at understanding and counteracting the pathogenic cascades induced by misfolded or aggregated proteins, the comprehension of the molecular bases of the mechanisms that lead to their formation and to the induced cell toxicity is still lacking. To this aim, the development of approaches that enable the characterization of protein aggregates in their natural environment and that provide a global insight in the physiological alterations induced by their formation is of huge importance. Noteworthy, in the 90’s one of the first direct evidence of the β-sheet structures in the β-amyloid plaque was provided *in situ* by synchrotron radiation (SR) Fourier transform infrared (IR) microspectroscopy (SR-µFTIR), analyzing the brain from an Alzheimer’s disease patient (Choo et al., 1996). This important result paved the way to other *in situ* studies that employed vibrational spectroscopies to shed light on the molecular bases of protein misfolding diseases, emerging as promising tools in the field of neurodegeneration.

IR spectroscopy holds a great deal of promise for studying *in situ* the amyloid structural properties and aggregation mechanisms. In particular, this vibrational tool enables the sample characterization in a label-free and non-destructive way, a characteristic that allows complementary analyses on the same sample, which does not require pre-analytical chemical modification. However, since the standard protocols for medical histopathology diagnosis include sample fixing, several works on infrared spectroscopy of fixed specimens are reported (Meuse and Barker, 2009). Indeed, fixation facilitates sample storage and could be useful to correlate routine histomorphologic microscopic examinations with the chemico-physical information obtained by IR spectroscopy. Nonetheless, several drawbacks of fixed sample characterization exist, because it has become evident that how the sample is handled prior to IR analysis is crucial to obtain reliable structural information, and particularly the interference due to external substances, such as dyes or fixatives or even culture media, has to be taken into consideration (Zohdi et al., 2015). In this perspective, to apply IR spectroscopy for the investigation of the structural properties of biological samples, it is imperative to evaluate how different methods of sample preparation could affect the IR spectra, and to analyze unfixed samples when possible, thus avoiding misleading interpretation of the spectral data.

Interestingly, numerous works are reported in literature, illustrating the ability of this vibrational approach to detect *in situ* changes in protein structural features and aggregate properties associated with different disorders. The possibility to highlight biochemical and structural changes directly in cells, in tissues or biofluids provides on one hand a tool for an early diagnosis of the disease, on the other the potential to get new insights into the mechanisms of amyloid formation and toxicity. Since the IR Amide I band is sensitive to fine protein structural details (Barth, 2007; Zandomeneghi et al., 2004; Seshadri et al., 1999; Sarroukh et al., 2013), representing a fingerprint of its conformational properties, IR spectroscopy has been used to characterize aggregation intermediates and amyloid fibrils. For instance, conventional FTIR spectroscopy (Surmacz-Chwedoruk et al., 2012; Bhak et al., 2014) and super-resolution IR imaging methods (Klementieva et al., 2020) have been employed to study amyloid polymorphism. Interestingly, isotope-edited IR has been applied to study conformational changes and aggregation or co-aggregation of two proteins copresent in the same sample (Natalello et al., 2016). Then, an interesting example of the application of IR spectroscopy at nanometer lateral resolution is related to the aggregation mechanism of the Josephin domain of ataxin-3 protein. The combination of atomic force microscopy and infrared spectroscopy allowed the identification of oligomeric intermediates sharing the structural properties of the native protein or being more similar to fibrils. These results indicate an aggregation process involving an initial formation of native-like assemblies, therefore suggesting a model of “first-aggregation-then-misfolding” for this protein (Ruggeri et al., 2015).

Notably, the possibility to characterize biofluids—including blood derivatives, saliva, urine and tears—could make it possible to develop a spectroscopic method for the early detection of neurodegenerative biomarkers in patient’ samples collected in a non-invasive way (Paraskevaidi et al., 2017). For instance, FTIR spectroscopy of blood plasma samples showed a decreasing trend in Alzheimer’s patients compared with healthy controls (HCs) for the ratios of lipid-to-protein, phosphate-to-carbohydrate, and RNA-to-DNA (Paraskevaidi et al., 2017). Moreover, El Khoury and others reported that the FTIR characterization of serum samples from patients affected by multiple sclerosis and amyotrophic lateral sclerosis (ALS) led to a satisfactory differentiation of the two pathologies (El Khoury et al., 2019). In particular, the Authors found that the most important spectral differences were ascribable to variations in the structure of DNA and RNA and to modifications of glycolipids, glycoproteins, and collagen (El Khoury et al., 2019). Recently, we applied vibrational spectroscopy supported by multivariate analysis for the characterization of tears—a very promising biofluid for diagnostic purposes—from patients affected by ALS and HCs. Particularly, an excellent discrimination of the two sample classes was obtained, and it was possible to disclose ALS spectroscopic markers related to protein and lipid alterations compared with HCs (Ami et al., 2021a).

It should be emphasized that these objectives are also achievable thanks to important advances in both instrumentation—that allows extending applications to spatially resolved analysis on nanometric scale—and in multivariate analysis methods that enable the investigation of big spectral data-set in a large variety of samples.

### Infrared Spectroscopy: From Micro- to Nano-Scale Lateral Resolutions

Infrared spectroscopy has a long history in biophysics, being extensively applied to the study of the secondary structure and aggregation of proteins *in vitro* (Barth, 2007; Natalello and Doglia, 2015; Seshadri et al., 1999; Sarroukh et al., 2013), as well as of the structural properties of lipids, carbohydrates, and nucleic acids (Casal and MantschKuhn, 1950; Sinclair at al., 1952a; Sinclair at al., 1952b; Zhizhina and Oleinik, 1972, 1984; Taillandier and Liquier, 1992; Kačuráková and Wilson, 2001). Particularly, IR spectroscopy allows to characterize the protein secondary structures mainly through the analysis of the Amide I band (1,700–1,600 cm^−1^), due to the CO stretching vibrations of the peptide bond, where *β*-sheets in native proteins and in protein aggregates display different components (Seshadri et al., 1999; Zandomeneghi et al., 2004; Barth, 2007; Sarroukh et al., 2013; Natalello and Doglia, 2015).

**FIGURE 1:**
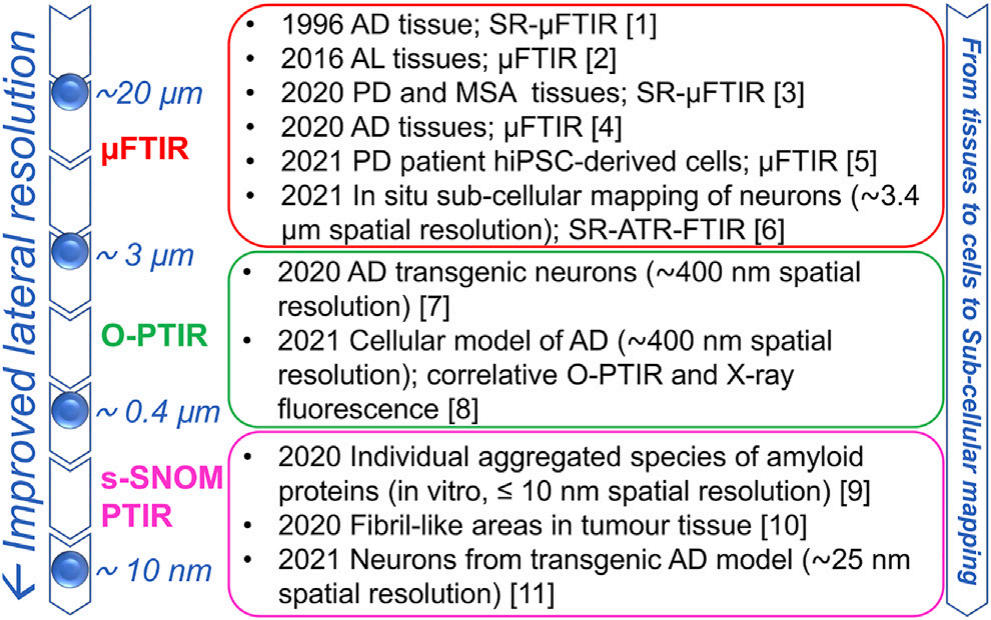
Infrared spectroscopy approaches: from the micro- to the nano-scale characterization of biological samples. The lateral resolutions and selected examples for the *in situ* study of protein aggregation are reported for different IR approaches. The increased resolution enables characterizing protein aggregates in complex systems from tissues, to intact cells and to sub-cellular areas. References: (1) Choo et al., 1996; (2) Ami et al., 2016; (3) Araki et al., 2020; (4) Röhr et al., 2020; (5) Gustavsson et al., 2021a; (6) Hartnell et al., 2021; (7) Klementieva et al., 2020; (8) Gustavsson et al., 2021b; (9) Waeytens et al., 2020 (10) Paluszkiewicz et al., 2020 (11) Freitas et al., 2021.

In the last few decades, supported by technological advancements that led to the improvement of infrared sources, optics, and detectors, the combination of microscopy with FTIR spectroscopy made it possible to extend the IR investigations to more complex biological systems, including intact cells and tissues, thus bringing about its renaissance in structural biology (Miller and Dumas, 2010). In particular, the possibility to study selected areas of the sample under investigation makes the µFTIR approach particularly useful for the analysis of systems characterized by an intrinsic heterogeneity, such as precisely biological systems. Moreover, depending on the instrument characteristics, diffraction-limited spatial resolution can be achieved, thus extending the application of this technique to cells and subcellular compartments (Figure 1). In particular, when the microscope is equipped with conventional IR source and detector, the spatial resolution is of about a few tens of microns per side. Then, with a focal plane array detector that enables not only to collect the IR absorption spectrum of the sample but also an IR chemical imaging, a sampling area of a few microns, near to the diffraction limit, can be measured with a good signal to noise ratio (Centrone, 2015). Furthermore, the use of a synchrotron IR light source, with a brightness of at least two orders of magnitude higher than that of a conventional thermal source, even allows achieving diffraction-limited spatial resolution with enhanced signal to noise ratio, making it possible to explore the IR spectra at the subcellular level. However, even under the best conditions, the employed long wavelength and the lack of high numerical aperture objectives limit the spatial resolution at about 3–10 µm in the mid-IR range and it is a function of the wavenumber employed in the µFTIR imaging (Centrone, 2015).

A submicrometer spatial resolution can, instead, be achieved by the new technique called optical photothermal infrared (O-PTIR) super-resolution imaging. In this technique, a pulsed IR tunable laser is focused onto the sample where the IR absorption causes a temperature increase that changes the refractive index. The IR absorption is locally detected as a change in sample reflectivity using a visible laser which is collinear with the IR beam. In this set-up it is the visible laser at a fixed wavelength that determines the spatial resolution. Under the optimal conditions, spatial resolution of about 1 µm–400 nm has been reported (Klementieva et al., 2020; Zhang et al., 2016).

Interestingly, to achieve a spectral resolution beyond the diffraction limit two main methods based on the coupling of IR spectroscopy with atomic force microscopy (AFM) have been developed: the scattering scanning near-field optical microscopy (s-SNOM) and the photothermal-induced resonance (PTIR) (Centrone, 2015). In the s-SNOM method, the measurement of the amplitude and phase of light scattered from the tip close to the sample makes it possible to obtain the IR absorption coefficient of a thin portion of the sample in proximity to the tip. Although this approach allows a nanometer (≈20 nm) resolution, it should be noted that the IR s-SNOM spectra are not directly comparable to the standard far-field IR spectra (Centrone, 2015).

In PTIR, the absorption of light from a pulsed, wavelength-tunable laser induces the sample thermal expansion that can be recorded by an AFM cantilever. In this case, the lateral resolution (which is in the order of 20–100 nm) depends on the tip size and on the sample thermomechanical properties (Centrone, 2015). Therefore, PTIR and O-PTIR provide an indirect measurement of the vibrational absorption using a different set-up, which results in different lateral resolution, penetration capacity and requirements of the sample conditions. Of note, both PTIR and O-PTIR spectra are expected to be directly comparable with those measured by traditional FTIR spectrometers (Centrone, 2015; Zhang et al., 2016; Klementieva et al., 2020).

### Multivariate Analysis of Complex Biological System Spectroscopic Data

IR (micro)-spectroscopy monitors the global biochemical composition of the sample under investigation; therefore, the resulting spectrum is a superposition of the vibrational signatures of the different sample biomolecules. Particularly when applied to investigate the response of living systems under diverse physiological and pathological conditions, spectral evaluation may become significantly complicated, also because in some cases minor differences may contain critical information (Ami et al., 2013; Morais et al., 2020). For this reason, the interpretation of the spectral results requires the support of appropriate multivariate analysis approaches able to tackle the study of high-dimensional data and to explore the whole spectral information simultaneously. Multivariate analysis creates a statistical model which describes the relationship between the spectra and the cellular response. In doing this, multivariate analysis also verifies the statistical significance of the results and pulls out the spectral components carrying the higher spectral variance and which are important in describing the underlying cellular conditions.

In the early decades of the 20th century, the pioneering work of R. A. Fisher contributed to the development of multivariate statistical methodology for applications in various fields, marking thus the beginning of research in multivariate analysis as a statistical technique for drawing inferences from multivariate data and opening up the doors to new knowledge through appropriate analyses of data (Radhakrishna Rao, 1992; Anderson, 1996; Rencher, 2002). Inspired by his work, multivariate analysis soon became popular, and especially applied to those systems which can be described using a large number of variables (i.e., biological systems), whose connections or associations need to be investigated. Then, in the last two decades, the increasing processing power of computers and the improvements in statistical algorithms allowed an impressive development of multivariate analysis methodologies (Baştanlar and Ozuysal, 2014) including discriminant analysis and class-modeling techniques where multiple spectral variables are analyzed simultaneously to discriminate and assign unknown samples to pre-defined classes (Morais et al., 2020; Zeng et al., 2021). Indeed, the rapid successful development of IR spectroscopy in biomedical research further highlighted the crucial role provided by advanced machine learning techniques, e.g., deep-learning neural networks of complex datasets to extract important information and visualize it in a readily interpretable form (Morais et al., 2020; Krafft et al., 2009; Marini et al., 2008).

### Selected Recent Examples: Exploiting the Potentialities of Infrared Spectroscopy for Amyloid Studies in Complex Systems

In the following, we will discuss a few selected works where the Authors applied IR spectroscopic methods to investigate protein aggregates, both *in vitro* and *in situ* within complex systems, with particular attention to amyloid aggregation.

Having the unique advantage of being a label-free method, spatially resolved FTIR microspectroscopy can provide information on the content and structure of different biomolecules simultaneously. This peculiarity enabled us to characterize not only amyloid deposits within unfixed tissues from patients affected by light chain (AL) amyloidosis, but also the biomolecules within or surrounding the protein aggregates. This possibility contributed to demonstrate a connection between lipid distribution and amyloidogenesis also in AL amyloidosis, further highlighting the presence of common features among different protein misfolding diseases (Ami et al., 2016). The possibility of investigating these properties *in situ* and at the individual level is priceless. Indeed, it makes it possible to relate the biophysical features of the fibrils to their specific pathogenic profile and to the histological properties of the amyloid plaques, thus contributing to fill the gap in the knowledge about the bases of amyloidosis at the individual level.

The fundamental role of the brain environment in determining the structural and pathological features of protein aggregates has been investigated in several diseases such as in Parkinson’s disease (PD) and multiple system atrophy (MSA). Of note, in both cases *α*-synuclein is the most abundant protein in the respective brain inclusions: Lewy bodies (LBs) in PD and glial cytoplasmic inclusions (GCIs) in MSA. Unfixed post-mortem brain sections from individuals with PD or MSA diagnosis have been analyzed by synchrotron microFTIR, which disclosed significantly higher *β*-sheet structures in LBs compared to GCIs. This result not only confirmed that *α*-synuclein deposits formed in the complex biological environment are enriched in *β*-sheet elements, as found for amyloid-like structures induced in the test tube, but also disclosed important differences in the main conformation of the proteins accumulated in the brain of these different synucleinopathies (Araki et al., 2020).

Notably, the possibility offered by FTIR microspectroscopy to analyze unlabeled brain tissue sections from AD cases, with a spatial resolution able to investigate the amyloid plaque substructures, has recently provided evidence of the conformational events associated with plaque developments. In particular, the Amide I band analysis of different plaque types indicated an increased content of *β*-sheet structures from diffuse plaques to compact and classic cored plaques. Moreover, an increased weight of the low-wavenumber *β*-sheet band (at 1,628 cm^−1^) in proportion to the high-wavenumber band (at 1,693 cm^−1^) was observed in mature plaques (Röhr et al., 2020). These results suggest the formation of A-beta fibrils with a parallel *β*-sheet conformation during plaque developments in the brain of AD patients, as previously described *in vitro*.

One of the drawbacks of the analysis of post-mortem brain samples is that extensive protein aggregation already occurred, making it difficult to characterize the early stage of the process and, of course, to translate IR spectroscopy in a diagnostic setting in the case of neurodegenerative diseases. These limitations have been recently addressed using human induced pluripotent stem cells (hiPSC) from familial and idiopathic PD patients (Gustavsson et al., 2021a). FTIR microspectroscopy analyses allowed the simultaneous study of the protein secondary structures, the lipid oxidation and the lipid chain length, enabling to correlate differences in the structure and composition of the main biomolecules with the peculiar intracellular environment induced by each patient genome (Gustavsson et al., 2021a). The validation of such results in large cohorts of patients could represent the starting point for the development of new tools for PD diagnosis and patient stratification towards personalized medicine.

A further advancement to improve the *in situ* investigation of protein aggregates has been recently described by Hartnell and others (Hartnell et al., 2021). Here, the potential of the synchrotron attenuated total reflection (ATR) FTIR technique to study macromolecular inclusions, such as protein aggregates, at sub-cellular level, has been reported. In particular, it has been shown that SR-ATR-FTIR mapping technique makes it possible to achieve a diffraction limited spatial resolution of a few microns, allowing the *in situ* characterization of sub-cellular protein aggregates in degenerating neurons of 20–30 μm in size. This important result opens the possibility to get new insights into the mechanism of protein aggregate formation in brain tissue (Hartnell et al., 2021). Overall, the merit of this technological advancement in the improvement of spatial resolution is that it offers the possibility to visualize at the sub-cellular level markers of cell physiology and disease pathology, an ongoing challenge in biomedicine.

Among the methodologies recently developed for improving spatial resolution, Klementieva and others (Klementieva et al., 2020) illustrated the application of O-PTIR spectroscopy to detect protein aggregates directly in neurons, at a sub-cellular level. O-PTIR, indeed, allows to tackle a very challenging task, that is to overcome the diffraction limit of IR light, providing a spatial resolution that is determined by the focusing visible laser achieving a few hundred nanometer resolution (Klementieva et al., 2020; Zhang et al., 2016).

By O-PTIR analysis on intact AD transgenic neurons, the Authors detected, for the first time, polymorphic protein aggregates at sub-cellular level, suggesting multiple Aβ aggregation mechanisms leading to assemblies with different structures and likely different neurotoxic effects (Klementieva et al., 2020).

Thanks to the recent technical advances of the O-PTIR instrumentations, this approach is also successfully applied to living cell imaging at sub-micrometer resolution. Interestingly, with this methodology Raman and IR spectra can be also collected simultaneously from the same sample area (Spadea et al., 2021).

Of note, very recently Gustavsson and others illustrated the application of O-PTIR spectroscopy combined to synchrotron-based X-ray fluorescence (S-XRF) to study amyloid aggregation processes in a cellular model of AD, at a sub-cellular level. In this way, it was possible to demonstrate directly in neurons the co-localization of iron clusters with elevated amyloid β-sheet structures and oxidized lipids (Gustavsson et al., 2021b).

Recent works that deserve mention illustrate the combination of two widely employed techniques for the characterization both *in vitro* (Waeytens et al., 2020) and *in situ* (Paluszkiewicz et al., 2020) of protein assemblies, namely atomic force microscopy and infrared spectroscopy. This method allows collecting images and infrared spectra at the nanometer scale, resulting, therefore, promising for the analysis of amyloid fibrils and other protein assemblies. Indeed, AFM-IR enables the correlation of the information contained in AFM images with the secondary structure and other information provided by the infrared spectra. As an *in situ* case study, in the AFM-IR analysis of the pleomorphic adenoma and the marginal tissue, fibril-like areas were observed in the AFM morphological characterization of the tumour tissue. Noteworthy, the AFM-IR approach enabled the collection of the IR spectra of the observed fibrils and of the surrounding area (Paluszkiewicz et al., 2020).

Moreover, the s-SNOM approach has been employed to measure individual cultured neurons from wild-type and transgenic AD model, enabling the identification of protein aggregates with a lateral resolution of ∼25 nm. The obtained results provided a strong mark that *β*-sheet rich structures associated with cell membranes can be sensed at a nanometer lateral resolution by this label-free method (Freitas et al., 2021).

Interestingly, the possibility to provide the global biochemical composition of the probed sample supported the extension of IR spectroscopy to the investigation of the molecular bases of amyloid toxicity. As an example, we exploited FTIR microspectroscopy, combined with multivariate analysis, to analyze *in situ* the spectral changes occurring in cultured intact HL-1 cardiomyocytes exposed to transthyretin (TTR)—wild type (WT) or the highly amyloidogenic variant L55P—in native or amyloid-like conformations (Ami et al., 2018). In particular, this study allowed us to detect the cell perturbations—affecting lipid bilayer fluidity/compactness and the cell metabolic/phosphorylation status—induced by the proteins in their different conformational states (Ami et al., 2018).

Furthermore, with the purpose of investigating possible perturbations induced on intact cells by intracellular amyloidogenic proteins, we applied FTIR microspectroscopy, coupled to multivariate analysis, to study *Escherichia coli* cells—taken as model system—expressing the human ataxin-3 (ATX3), both a physiological (ATX3-Q24) and a pathological ATX3 variant (ATX3-Q55) (Ami et al., 2021b). Notably, by the FTIR analysis it was possible not only to monitor the protein aggregation, but also the induced cell perturbations. In particular, we found that the toxic oligomeric species associated with the expression of the pathological variant ATX3-Q55 were responsible for the main spectral changes, mostly ascribable to the cell envelope modifications, a result that was also supported by electron microscopy analysis (Ami et al., 2021b).

## Conclusion and Future Perspectives

The growing interest in the application of IR spectroscopy in different fields of biological and biomedical research is accompanied by an ongoing technological advancement that makes it possible to extend the IR investigations to more complex biological systems. In particular, the development of more and more sophisticated methodologies, allowing to achieve or even to overcome diffraction-limited spatial resolution, enables the exploration of the IR response at sub-micrometer scale. This is a crucial result to obtain more detailed structural information on protein aggregates in intact cells and tissues, making IR spectroscopy a powerful tool for basic research as well as for clinical purposes. The possibility to characterize the conformational features of protein aggregates in their natural microenvironment has the potential to help us narrow the gap between our knowledge of amyloid aggregation mechanisms *in vitro* and *in vivo* (Faravelli et al., 2022).
